# Provenancing antiquarian museum collections using multi-isotope analysis

**DOI:** 10.1098/rsos.220798

**Published:** 2023-02-08

**Authors:** Samantha Neil, Jane Evans, Janet Montgomery, Rick Schulting, Chris Scarre

**Affiliations:** ^1^ School of Archaeology, University of Oxford, Oxford OX1 ETG, UK; ^2^ National Environmental Isotope Facility, British Geological Survey, Keyworth, Nottinghamshire, UK; ^3^ Department of Archaeology, Durham University, Durham, UK; ^4^ Durham University, Durham, UK

**Keywords:** isotopes, biogeochemistry, neolithic

## Abstract

Many of the most significant archaeological sites in Europe were excavated by antiquarians over one hundred years ago. Modern museum collections therefore frequently contain human remains that were recovered during the nineteenth and early twentieth centuries. Here we apply multi-isotope analysis (^87^Sr/^86^Sr, *δ*^18^O, *δ*^13^C, *δ*^15^N) and ^14^C dating to evaluate the provenance of human remains within a collection that is thought to have been recovered from one of the most important archaeological sites in Britain. Excavated in 1910, the site of Coldrum in Kent is a megalithic burial monument that may be one of the earliest sites associated with the transition to farming in Britain. The interpretation of this site is therefore key to understanding how agriculture began. Using isotope analysis we show that although the human skeletal collections attributed to Coldrum do contain some of the earliest dated Neolithic human remains in Britain, they also contain the remains of individuals of fifth to seventh centuries AD date. We evaluate subsistence and mobility patterns of early Neolithic populations and provide new information about the origins of those individuals in the collection that date to the fifth to seventh centuries AD. We demonstrate the utility of employing isotope analysis to provide direct and independent information about the provenance of human remains in museum collections.

## 1. Introduction

Some of the most important archaeological sites were excavated more than a century ago. Remains recovered during antiquarian investigations, therefore, form a significant component of museum collections and provide the basis for study and understanding of the past today. An example of this is the site of Coldrum, Kent. It is a megalithic burial monument, with a substantial stone chamber that was found to contain human remains. The practice of building monuments of this type was introduced to Britain from the European mainland during the transition to farming, the development of the Neolithic, in the early fourth millennium BC. As well as being associated with the introduction of new traditions of burial and monument construction, this period also saw a radical change in subsistence patterns. New technology, such as pottery production and new species of non-native plants and animals, such as domesticated cereals, cattle and sheep, were also introduced from the European mainland at this time [1,2]. Many authors suggest that Coldrum long barrow could be one of the earliest monuments to have been constructed during the start of the Neolithic, and the Coldrum collection has therefore become central to the debate over how farming began in Britain [3–7].

Excavations began at the monument in 1910, following reports of the discovery of pottery and skeletal remains at the site [8–10]. The human remains that were found during this excavation were given to the Royal College of Surgeons (RCS) in London [11]. Established in the eighteenth century, the RCS housed one of the largest and most geographically diverse antiquarian collections in Europe [12].

Investigations at Coldrum resumed in the 1920s and further human remains were recovered: these are also recorded as having been given to the RCS [13]. After the war, the cranial remains, fragments of cranial vault and mandibles in the collection were moved from the RCS to the University of Cambridge, where accession records document their arrival in 1950–51. All the post-cranial remains attributed to the site were transferred to the Natural History Museum in London. In addition to the collections that are currently held by these two institutions, a small number of human remains, also attributed to Coldrum, are curated by Maidstone Museum.

The Coldrum collection, therefore, has a complex history: the human remains are fragmentary and disarticulated and they have been moved between and curated by multiple institutions. All the remains in these collections are attributed to the Coldrum excavations by the institutions that curate them. However, only a few specimens were directly labelled at the time of the excavations: only four fragments of cranial vault have original labelling that means they can be securely associated with their context of excavation at the site. F.J. Bennett's account of the excavations describes how human remains were recovered from two different levels or ‘platforms’ inside the megalithic burial chamber: from an upper level (platform 1) and lower level (platform 2) [10]. The four cranial vault fragments with original labelling can be attributed to two individuals from the upper level and two individuals from the lower level of Bennett's excavations. The two cranial fragments found on the lower level have produced some of the earliest dates yet obtained on human remains associated with a Neolithic monument in Britain, dating to the first two centuries of the fourth millennium BC [14]. The two fragments found on the upper level have dates that fall later in the fourth millennium BC. It is therefore hypothesized that there were at least two different phases of burial activity at the site: Bayesian modelling suggests that the first phase of activity began in *3980–3800 cal BC* (*95% probable*) or *3960–3880 cal BC* (*68% probable*) and ended in *3930–3750 cal BC* (*95% probable*) or *3910–3770 cal BC* (*68% probable*). The subsequent phase of activity is postulated to have begun in *3730–3540 cal BC* (*95% probable*) or *3660–3570 cal BC* (*68% probable*) and ended in *3320–3010 cal BC* (*95% probable*) or *3330–3170 cal BC* (*68% probable*; Model 2 by Wysocki *et al.*, re-calibrated using IntCal20 and OxCal 4.4) [14–16].

As the Coldrum collection has been shown to contain some of the earliest human remains attributable to a Neolithic monument in Britain [6,7,14], it is critical to interpretation of the transition to farming which occurred at the start of the fourth millennium BC [3–7]. Here we conduct ^14^C dating of human remains in the collection that have not previously been radiocarbon dated in conjunction with ^87^Sr/^86^Sr, *δ*^18^O, *δ*^13^C analysis of tooth enamel to provide direct information about where individuals obtained their childhood diet. We combine this with *δ*^15^N and *δ*^13^C analysis of dental collagen to provide direct information about childhood dietary composition.

## 2. Methods

### 2.1. ^87^Sr/^86^Sr analysis

Application of strontium isotope analysis for geographical provenancing is based on the principle that ^87^Sr/^86^Sr varies according to the age, initial Sr/Rb ratio and geological history of bedrock [17,18]. Strontium weathers out from rocks, into soils and ground waters where it becomes bioavailable, being incorporated into plants and, in turn, into animals [19]. Conventionally measured ^87^Sr/^86^Sr values do not vary significantly between trophic levels and because enamel does not remodel once formed and is highly resistant to diagenesis, ^87^Sr/^86^Sr values directly reflect the sources of dietary strontium to which individuals were exposed during tooth formation [20–22]. Comparison of the isotope ratios preserved in tooth enamel to mapped bioavailable ranges in plants and water can therefore be used to evaluate where an individual obtained their diet [23–25]. Here, we analyse ^87^Sr/^86^Sr values in adjacent consecutively mineralizing molar teeth (first, second and third permanent molars), where present, to compare isotope ratios in teeth that form at different stages of early life. Formation of the first permanent adult molar crown commences *in utero*, just prior to birth [26], and completes by approximately 4.5 ± 0.5 years of age; formation of the second molar crown occurs between approximately 2.5 ± 0.5 years and 8.5 ± 0.5 years of age; the timing of third molar formation is most variable with initial cusp formation from approximately 8.5 ± 0.5 years of age and crown completion by approximately 14.5 ± 0.5 years [27,28].

### 2.2. *δ*^18^O_carbonate_ and *δ*^13^C_carbonate_ analysis

There are two ionic forms of oxygen in tooth enamel that are suitable for oxygen isotope analysis, structural carbonate (${\mathrm{CO}}_{3}^{2\text{–}}$, *δ*^18^O_carbonate)_ and phosphate (${\mathrm{PO}}_{4}^{3\text{–}}$, *δ*^18^O_phosphate)_ [29,30]. Here we use oxygen and carbon isotope analysis of the ${\mathrm{CO}}_{3}^{2\text{–}}$ fraction of enamel. ^18^O/^16^O varies geographically with climate, according to factors such as temperature, continentality, latitude and altitude [31,32].

Use of oxygen isotope analysis for geographical provenancing is based on the principle that *δ*^18^O values in tooth enamel of mammals who are obligate drinkers reflect the oxygen isotope composition of drinking water [33–36]. However, in human populations, the oxygen isotope composition of ingested fluids can also be influenced by culturally mediated behaviour, for example, consumption of fluids that have undergone fractionation through biological processes, such as cow's milk [37–40] or breast milk [41]. Enamel that forms while an infant is being breastfed (e.g. deciduous molars or first molars) may therefore record higher *δ*^18^O values than that which forms later in childhood [42,43]. Culinary practice, such as boiling water, brewing or stewing food, has also been shown to influence the oxygen isotope composition of ingested fluids [44]. In addition to the variation in *δ*^18^O composition of ingested fluids that may be generated by culinary practices, recent study of enamel from modern human populations suggests that *δ*^18^O values can also vary by up to approximately 2‰ between samples taken at the same location in antimeres of teeth from the same individual [45]. Such variability in *δ*^18^O values contrasts with that of ^87^Sr/^86^Sr values, which have been to shown to vary only in the fourth decimal place between samples taken from the same location from antimeres of teeth, by up to 0.000192 [45]. It has also been shown that human populations buried in adjacent regions of temperate Europe can have similar *δ*^18^O values, as values in local precipitation overlap [46]. As a consequence of the above factors, oxygen isotope analysis of enamel from human populations is useful for making broader scale distinctions, for differentiating individuals who originate from regions of much colder climate and have very low *δ*^18^O_carbonate_ values, (<24.5‰, or *δ*^18^O_phosphate_ VSMOW values < 15.5‰) from those who sourced their childhood dietary resources from temperate locations e.g. [47–53].

### 2.3. Laboratory procedures: strontium and oxygen isotope analysis

This study sampled previously undated dentition in mandibular remains from a total of ten individuals. The skeletal remains in the collections that are attributed to Coldrum are disarticulated and fragmentary. To avoid sampling fragments of dentition that could belong to the same individual, samples were only taken from right-sided mandibular dentition. Teeth were only sampled from the left side when the mandible was complete. Loose teeth and maxillary dentition were not sampled.

In addition to sampling mandibles attributed to Coldrum in the University of Cambridge Duckworth Laboratory collection that have been allocated accession numbers (EU.1.5.127 and EU.1.5.129 to EU.1.5.133), three further unlabelled specimens in the Duckworth collection that are attributed to Coldrum but have not been allocated accession codes were also sampled and given codes for the purpose of this study: COL/UN8, COL/UN and COL/UNBOYD, where UN stands for ‘unidentified’. Specimen COL/UNBOYD has the letters ‘Boyd’ handwritten directly on the side of the mandible. The number ‘8’ is handwritten on specimen COL/UN8. In addition to analysis of teeth from these mandibular remains held at the University of Cambridge, dentition from one mandible fragment curated by Maidstone Museum (accessioned as specimen 6) was also sampled.

An enamel sample of approximately 10 mg in weight was taken from the crown of each tooth for oxygen isotope analysis and another of 20–30 mg in weight was taken for strontium isotope analysis. As analysis was conducted on bulk enamel, isotope ratios represent the weighted average of all sources of dietary strontium to which the individual was exposed at the time of tooth formation [21]. It has been shown that once enamel is fully mineralized it is highly resistant to diagenesis and retains *in vivo* ^87^Sr/^86^Sr values e.g. [54–56]. However, caries may alter the *in vivo* isotope ratios: [45] none of the teeth sampled in this study exhibited any evidence of caries and all were completely mineralized.

Following procedures described in Montgomery 2002 [54], surface enamel was thoroughly abraded using a tungsten carbide dental burr. Enamel chips were cut using a flexible diamond-edged rotary saw and were then again mechanically cleaned using a tungsten carbide dental burr to remove any adhering dentine. Dental saws and burrs were cleaned ultrasonically for 5 min and rinsed three times in high purity de-ionized water between preparation of samples. Enamel samples were taken in clean sealed containers to the Class 100, HEPA-filtered laboratory facilities at the National Environmental Isotope Facility (NEIF, Keyworth, Nottingham, England). Here, they were cleaned ultrasonically and rinsed in high purity water (Millipore Alpha Q). They were then dried and weighed into pre-cleaned Teflon beakers. To obtain strontium concentrations they were spiked with a known amount of ^84^Sr tracer solution. Each sample was dissolved in Teflon distilled 8 M HNO_3_. Samples were converted to chloride using 6 M HCl, taken up in titrated 2.5 M HCl and pipetted onto ion-exchange chromatography columns. Strontium was separated with Eichrom AG50-X8 resin (200–400 mesh). Procedural blanks were below 80 pg.

Samples were then loaded on to Re filaments using a method adapted from Birck 1986 [57]. Strontium isotope composition and concentrations were determined by Thermal Ionization Mass Spectrometry using a ThermoTriton automated multi-collector mass spectrometer. To correct for fractionation during the process of mass spectrometry, ^87^Sr/^86^Sr values were normalized to the accepted value for ^88^Sr/^86^Sr = 0.1194. During the period of this study, the machine gave a value for the international standard for ^87^Sr/^86^Sr (NBS 987) of 0.710253 ± 0.000012 (2*σ*, *n* *=* 350). An estimate of the reproducibility of strontium concentration (Sr ppm) was provided by replicate analysis of an aliquot of bone standard solution (NIST1486), which gave 7.22 ± 0.27 ppm (±3.75%, 1*σ*, *n* *=* 16).

Initial preparation of enamel samples for *δ*^18^O and *δ*^13^C analysis was conducted using the same methods used above for strontium isotope analysis. Cleaned enamel chips were powdered using a pestle and mortar at the National Environmental Isotope Facility. Oxygen (*δ*^18^O_carbonate_) and carbon (*δ*^13^C_carbonate_) isotope ratios in the carbonate fraction of enamel were then determined with a GV IsoPrime dual inlet mass spectrometer, using approximately 3 mg of the clean powdered enamel, following the method described in Chenery *et al*. 2012 [29]. Isotope ratios are reported as delta (*δ*) values in per mil (‰) relative to the *δ*^18^O_VPDB_ scale calculated using an in-house carbonate reference material, Keyworth Carrera Marble (KCM), that has known *δ*^13^C_VPDB_ (2.00‰) and *δ*^18^O_VPDB_ (−1.73‰) having been measured against IAEA-603 (*δ*^18^O_VPDB_ = −2.37‰) and NBS 19 (*δ*^18^O_VPDB_ = −2.20‰). Calibration of the dual inlet isotope ratio mass spectrometer using NBS19 and NBS18 (*δ*^18^O_VPDB_ = −23.01‰) shows a consistent but small scaling factor for *δ*^18^O equivalent to approximately 0.2‰ over the approximately 20‰ range between NBS19 and NBS18, which is negligible given the proximity of enamel *δ*^18^O_carbonate_ data to NBS19, external precision on sample data, uncertainty associated with the assigned value of NBS18, and overall sample reproducibility.

Analytical reproducibility during measurement of this run of samples was ± 0.4‰ (1*σ*, *n* *=* 15) for *δ*^13^C and ± 0.11‰ for *δ*^18^O (1*σ*, *n* *=* 15). Enamel samples were also run in duplicate to assess the replicability of *δ*^18^O_carbonate_, *δ*^13^C_carbonate_ values: *δ*^18^O values varied by a mean of 0.2 ± 0.3‰ (1*σ*, *n* *=* 6) and 0.2 ± 0.2‰ (1*σ*, *n* *=* 6) for *δ*^13^C_carbonate_ between duplicates. *δ*^18^O_carbonate_ values have been normalized to the VSMOW scale using the equation of Kim *et al.* [58] (*δ*^18^O_VSMOW−SLAP_ = 1.03092 × *δ*^18^O_VPDB_ + 30.92‰).

To enable comparison of these results to data from studies that have sampled the phosphate (${\mathrm{PO}}_{4}^{3\text{–}}$) fraction e.g. [59,60], conversion from *δ*^18^O_carbonate_ to *δ*^18^O_phosphate_ is undertaken using the regression equation of Chenery *et al.* [29] (*δ*^18^O_phosphate_ = 1.0322 × *δ*^18^O_carbonate_ – 9.6849). *δ*^18^O values of the *δ*^18^O_phosphate_ and *δ*^18^O_carbonate_ fractions are well correlated and the error involved in calculating *δ*^18^O_phosphate_ using this equation is low (0.28‰, 1*σ*) [29].

### 2.4. ^14^C dating and *δ*^15^N and *δ*^13^C_collagen_ analysis

With the exception of specimen 6 from the Maidstone Museum collection (see below) all radiocarbon dates and *δ*^15^N and *δ*^13^C_collagen_ results were obtained from collagen extracted from the roots of the same permanent first molar teeth as those that were sampled for ^87^Sr/^86^Sr, *δ*^18^O, *δ*^13^C_carbonate_ analysis. *δ*^15^N and *δ*^13^C_collagen_ values were measured in samples of bulk collagen taken from the roots of first permanent molars. The roots of first permanent molar teeth begin to form at approximately 3.5 ± 0.5 years; their formation is complete by approximately 7.5 ± 0.5 years of age [27].

Radiocarbon dating was conducted at the Oxford Radiocarbon Accelerator Unit (ORAU). Collagen was extracted using gelatinization and ultrafiltration techniques as described in Bronk Ramsey *et al.* [61] and Brock *et al.* [62,63]. Once samples were graphitized, isotope ratios were measured by accelerator mass spectrometry (AMS) using methods described in Dee and Bronk Ramsey [64] and Bronk Ramsey *et al.* [61]. Radiocarbon dates are calibrated using IntCal2020 and OxCal4.4 [15,16].

Use of *δ*^15^N and *δ*^13^C analysis to study dietary composition exploits the large natural variation in *δ*^13^C values of plants that use different (C_3_ or C_4_) photosynthetic pathways and the contrast in *δ*^13^C values between terrestrial C_3_ and marine ecosystems [65,66]. *δ*^13^C and *δ*^15^N values also vary geographically with environment and climate, according to temperature and water availability e.g. [67,68], with terrestrial species in northern Europe having lower *δ*^13^C and higher *δ*^15^N values than those in southern Europe, e.g. [69,70]. When dietary protein intake is sufficient, *δ*^13^C_collagen_ values predominately reflect the protein component of the diet, whereas *δ*^13^C_carbonate_ values in bioapatite reflect the isotope composition of the diet as a whole, including carbohydrates and lipids [71,72]. Carbon isotope analysis of bioapatite can therefore be particularly useful for elucidating the contribution of C_4_ plants to the diet, which may be under-represented when analysing *δ*^13^C_collagen_ e.g. [73,74]. This would not, however, be relevant for individuals dated to the fourth millennium BC in Europe, as the consumption of C_4_ plants such as millet is not known to have occurred here until the sixteenth century BC [75].

*δ*^15^N and *δ*^13^C_collagen_ analysis was conducted specifically for the purpose of dietary isotope analysis at the University of Bradford. Isotope measurements were made using a Thermo Flash EA 1112, with separated N_2_ and CO_2_ being introduced to a Delta plus XL via a Conflo III interface. Results are reported as delta (*δ*) values, per mil (‰), expressed relative to the international standard used for carbon isotope analysis Vienna Pee Dee Belemnite (VPDB) and for nitrogen, Ambient Inhalable Reservoir (AIR) [76]. International laboratory standards IAEA-600, IAEA-CH3, IAEA-CH6, IAEA-CH7, IAEA-N1 and IAEA-N2 were interspersed with samples in the analytical run. Internal standards were also used in the run: fish gelatine with a *δ*^15^N value of 14.4‰ and a *δ*^13^C value of −15.5‰ and bovine liver standard with a *δ*^15^N value of 7.6 ± 0.25‰ and a *δ*^13^C value of −21.6 ± 0.25‰. Analytical error as determined from repeated measurement of both international and internal standards during these runs of samples was 0.2‰ at one standard deviation for both *δ*^15^N and *δ*^13^C_collagen_. Every collagen sample in this study was also measured in duplicate (table 1), with the exception of sample COL/UN8, where analysis of the duplicated sample failed during the run. *δ*^15^N values varied between duplicated samples by a mean of 0.1 ± 0.1‰ (1*σ*, *n* *=* 9) and by a mean of 0.1 ± 0.1‰ (1*σ*, *n* *=* 9) between duplicates for *δ*^13^C_collagen_ (table 1). Where duplicates of samples were measured for *δ*^15^N, *δ*^13^C_collagen_, *δ*^18^O_carbonate_ and *δ*^13^C_carbonate_, the values cited in the results and discussion below are the mean of the two measurements. C:N ratios are also provided in table 1 and these are within the accepted ranges for collagen [77].

**Table 1.** Results of ^87^Sr/^86^Sr, *δ*^18^O, *δ*^13^C analysis of tooth enamel**,** radiocarbon dating and *δ*^15^N and *δ*^13^C analysis of collagen from specimens in the Coldrum collection. Age at death estimated based on stage of tooth development, following AlQahtani *et al*. [27]. Individuals with fully developed third molar tooth roots at the time of death classified as ‘adult’.

museum accession number	age	tooth	radiocarbon laboratory code	radiocarbon age (BP)	calibrated date (95% confidence) IntCal20, OxCal4.4	*δ*^13^C_collagen_	*δ*^15^N	C:N ratio	*δ*^13^C_collagen_ Duplicate	*δ*^15^N Duplicate	C:N ratio Duplicate	*δ*^13^C_collagen_ Average	*δ*^15^N Average	^87^Sr/^86^Sr	Sr (mg kg^−1^)	*δ*^13^C enamel carbonate ‰ VPDB	*δ*^18^O enamel carbonate ‰ VSMOW	*δ*^13^C enamel carbonate ‰ VPDB Duplicate	*δ*^18^O enamel carbonate ‰ VSMOW Duplicate	average *δ*^13^C enamel carbonate ‰ VPDB	average *δ*^18^O enamel carbonate ‰ VSMOW
EU.1.5.130	adult	LM1	OxA-28201	1529 ± 23	464–603 AD	−19.8	10.96	3.19	−19.81	10.7	3.19	−19.81	10.83	0.70924	72.9	−14.41	28.14			−14.41	28.14
		LM2												0.70912	55.6	−14.33	27.52	−14.25	27.77	−14.29	27.64
		LM3												0.70901	88.4	−13.93	27.03			−13.93	27.03
EU.1.5.131	adult	LM1	OxA-28202	1601 ± 23	420–539 AD	−20.28	10.93	3.33	−20.27	10.69	3.3	−20.28	10.81	0.70929	107.9	−16.09	28.15	−16.02	28.09	−16.05	28.12
		RM2												0.70914	112.5	−14.45	27.31			−14.45	27.31
EU.1.5.132	adult	RM1	OxA-28273	1480 ± 22	560–641 AD	−20.84	10.84	3.2	−20.73	10.87	3.18	−20.78	10.85	0.70907	57.0	−14.54	26.78	−13.93	27.53	−14.24	27.16
EU.1.5.133	adult	RM1	OxA-28272	1568 ± 23	430–561 AD	−19.93	9.94	3.31	−19.75	9.75	3.27	−19.84	9.84	0.70966	66.0	−15.92	26.69			−15.92	26.69
		RM2												0.70979	36.0	−13.65	27.30			−13.65	27.30
EU.1.5.127	adult	LM1	OxA-28203	5048 ± 28	3953–3773 BC	−21.35	8.52	3.21	−21.38	8.56	3.2	−21.37	8.54	0.70973	72.0	−15.24	27.33			−15.24	27.33
		LM2												0.70981	72.1	−14.71	27.00			−14.71	27.00
		LM3												0.70992	58.3	−13.95	26.93	−13.90	27.00	−13.93	26.97
COL/UN8	sub-adult >12.5 years	LM1	OxA-28204	5119 ± 30	3984–3801 BC	−21.18	8.99	3.22	None	None	None	−21.18	8.99	0.70831	54.2	−15.70	27.10			−15.70	27.10
		LM2												0.70949	51.1	−15.16	27.13	−15.11	27.14	−15.13	27.13
EU.1.5.129	adult	RM1	OxA-28205	5093 ± 28	3966–3798 BC	−20.38	9.02	3.21	−20.46	9.13	3.21	−20.42	9.08	0.70997	51.2	−14.72	26.79			−14.72	26.79
		RM2												0.71029	33.9	−14.99	26.89			−14.99	26.89
COL/UN	adult	RM1	OxA-28206	5136 ± 29	4040–3804 BC	−21.35	8.52	3.21	−21.38	8.56	3.21	−21.37	8.54	0.70889	53.0	−15.95	27.11			−15.95	27.11
		RM2												0.70795	36.0	−15.45	26.85			−15.45	26.85
		RM3												0.70892	45.6	−15.81	26.63	−15.87	26.45	−15.84	26.54
COL/UNBOYD	adult	RM1	OxA-28207	5048 ± 29	3954–3770 BC	−21.73	8.28	3.24	−21.79	8.43	3.26	−21.76	8.36	0.70930	59.0	−16.65	27.10			−16.65	27.10
Maidstone Museum specimen 6	child, c. 18 months at death	DP2	OxA-28200	4550 ± 27	3371–3103 BC	−21.23	12.38	3.2	−21.09	12.28	3.22	−21.16	12.33	0.70956	108.0	−16.99	27.87			−16.99	27.87

The mandible from Maidstone Museum (specimen 6) is from a child, aged approximately 18 months at death. A sample of mandibular bone was taken for radiocarbon dating and *δ*^15^N and *δ*^13^C_collagen_ values were also obtained from this. ^87^Sr/^86^Sr, *δ*^18^O_carbonate_ and *δ*^13^C_carbonate_ values were obtained by sampling enamel from the left deciduous second molar tooth of this individual.

## 3. Results

**Figure 1.**
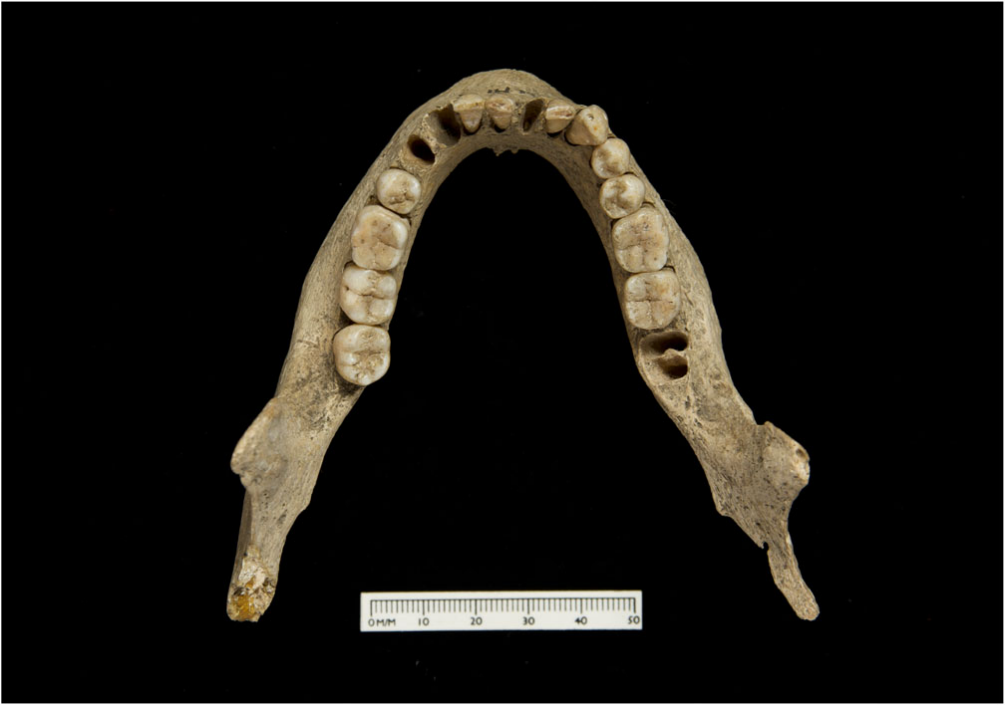
Photograph of individual EU.1.5.130 from the Coldrum collection. The individual is dated to 464–603 AD (OxA-28201; 95% confidence, IntCal20, OxCal4.4).

**Figure 2.**
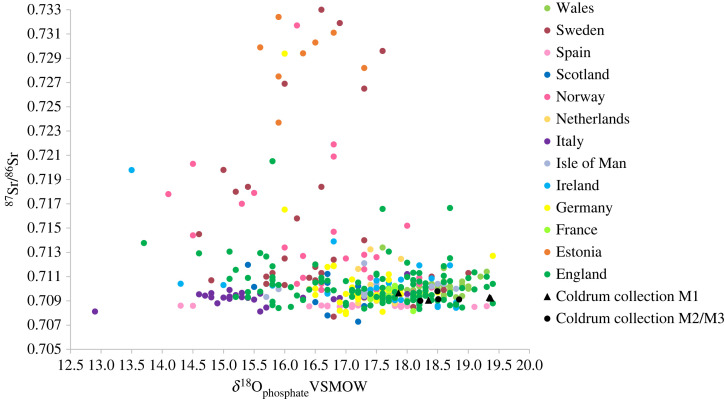
*δ*^18^O_carbonate_ (VSMOW) versus ^87^Sr/^86^Sr of enamel from individuals of fifth to seventh centuries date in the Coldrum collection in comparison to post-infant dentition of Early Medieval populations buried in Europe (fifth to tenth centuries AD) [78]. Triangles = permanent first molar teeth. Circles = post-infant dentition: permanent second molar, third molar and premolar tooth crowns that form later in childhood; *δ*^18^O values are therefore unlikely to be influenced by breastfeeding.

Of the 10 sampled specimens, four in the collection attributed to Coldrum date to the fifth to seventh centuries AD (EU.1.5.130, EU.1.5.131, EU.1.5.132 and EU.1.5.133; figure 1). ^87^Sr/^86^Sr values of enamel from these individuals range between 0.7090 and 0.7098 (mean 0.7093 ± 0.0003, 1*σ*, *n* = 8); Sr concentrations range between 36 and 113 ppm (mean 75 ± 25 ppm, 1*σ*, *n* = 8; figure 2). *δ*^18^O_carbonate_ values of samples of first permanent molar enamel from these individuals range between 26.7 and 28.1‰ (mean 27.5 ± 0.7‰, 1*σ*, *n* = 4). The mean enamel *δ*^18^O_carbonate_ value of enamel from first permanent molar teeth is 0.2‰ higher than that of enamel from second and third permanent molar teeth, where values range between 27.0 and 27.6‰ (mean of 27.3 ± 0.25‰, 1*σ*, *n* = 4). Enamel *δ*^13^C_carbonate_ values of all teeth sampled in the group range that date to the fifth to seventh centuries AD range between −16.1 and −13.7‰ (mean −14.6 ± 0.9‰, 1*σ*, *n* = 8). *δ*^15^N values of first molar root dentine samples range between 9.8 and 10.9‰ (mean 10.6 ± 0.5‰, 1*σ*, *n* = 4) and *δ*^13^C_collagen_ values of these samples range between −20.8 and −19.8‰ (mean −20.2 ± 0.5‰, 1*σ*, *n* = 4).

**Figure 3.**
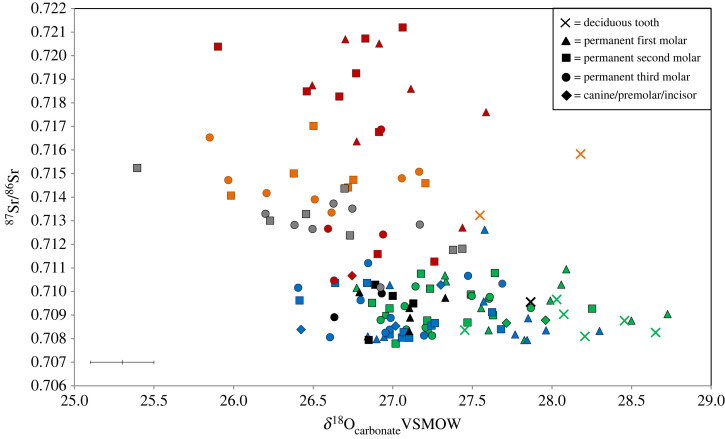
^87^Sr/^86^Sr versus *δ*^18^O_carbonate_ (VSMOW) values of enamel from dentition of Neolithic date attributed to Coldrum, in comparison to individuals excavated from other sites of earlier Neolithic date in Britain [79–82]. Tooth types illustrated within the diagram. Coldrum collection = Black symbols; Whitwell cairn = Red symbols; Penywyrlod long cairn = Orange symbols; Ty Isaf long cairn = Grey symbols; Hazleton North long cairn = Blue symbols; Hambledon Hill long barrow and causewayed enclosures = Green symbols. Analytical error for *δ*^18^O_carbonate_ is shown as ± 0.2‰ (2*σ*). Error for ^87^Sr/^86^Sr is within the symbol.

Five individuals in the sampled collections date to the forty-first to thirty-eighth centuries BC (EU.1.5.127, EU.1.5.129, COL/UN8, COL/UN and COL/UNBOYD; figure 3). ^87^Sr/^86^Sr values of enamel from these individuals range between 0.7079–0.7103 (mean 0.7093 ± 0.0007, 1*σ*, *n* = 11). Sr concentrations in enamel from this group range between 34 and 72 ppm (mean 53 ± 12 ppm, 1*σ*, *n* = 11). *δ*^18^O_carbonate_ values of enamel samples from the first permanent molar teeth of these individuals range between 26.8 and 27.3‰ (mean 27.1 ± 0.2‰, 1*σ*, *n* = 5). The mean *δ*^18^O_carbonate_ value of enamel from first permanent molars is again 0.2‰ higher than values in enamel from second and third permanent molar teeth, where values range between 26.5 and 27.1‰ (mean 26.9 ± 0.2‰, 1*σ*, *n* = 6). Individuals in the group that date to the forty-first to thirty-eighth centuries BC have enamel *δ*^13^C_carbonate_ values that range between −16.7 and −13.9‰ (mean −15.3 ± 0.7‰, 1*σ*, *n* = 11). *δ*^15^N values of first molar root dentine samples from these individuals range between 8.4 and 9.1‰ (mean 8.7 ± 0.3‰, 1*σ*, *n* = 5) and *δ*^13^C_collagen_ values of these samples range between −21.8 and −20.4‰ (mean −21.2 ± 0.5‰, 1*σ*, *n* = 5).

The specimen (number 6) from Maidstone Museum dates to the thirty-fourth to thirty-second centuries BC, with a calibrated radiocarbon date of 3369–3106 BC (OxA-28200; table 1; figure 3). Enamel from the second deciduous molar tooth of this individual, a child aged approximately 18 months at death, gave an ^87^Sr/^86^Sr value of 0.7096, with a strontium concentration of 108 ppm. Enamel from the same tooth gave an *δ*^18^O_carbonate_ value of 27.9‰ and an *δ*^13^C_carbonate_ value of −16.9‰. Collagen sampled from the mandible of this individual gave an *δ*^15^N value of 12.3‰ and a *δ*^13^C_collagen_ value of −21.2‰.

## 4. Discussion

Although the Coldrum collection contains human remains dated to the fourth millennium BC, it also contains individuals of fifth to seventh century AD date. Accessions documents at the University of Cambridge state that all these remains are attributed to excavations at the site of Coldrum and that they were moved to the Duckworth Laboratory from the Royal College of Surgeons (RCS) in 1950–51.

The Coldrum burial monument with its stone chamber and long mound is of a style of construction that is associated with the earlier fourth millennium BC and no Anglo-Saxon material culture is known from the site. The possibility that Coldrum was reused for burial in the Early Medieval period might perhaps be considered. Some 27 other Neolithic long barrows and chambered tombs are documented as having been reused for burial during the early Anglo-Saxon period in Britain, with examples including Lyneham long barrow, Oxfordshire [83–85]. Alternatively, the possibility that the individuals of fifth to seventh centuries AD date do not derive from Coldrum should be considered. The Coldrum collection has a complex curatorial history, having been transferred between and curated by several different institutions in the century following its excavation. During the nineteenth and early twentieth centuries the Royal College of Surgeons housed one of the largest and most diverse antiquarian collections in Europe [12]. However, both the RCS collections and the buildings in which they were held were extensively damaged during the Second World War and the collections were subsequently dispersed to other institutions following the war, including the Natural History Museum and the University of Cambridge (ibid.) The possibility that human remains from Coldrum were mixed with those from other sites before being transferred to the University of Cambridge following the war cannot be excluded.

**Figure 4.**
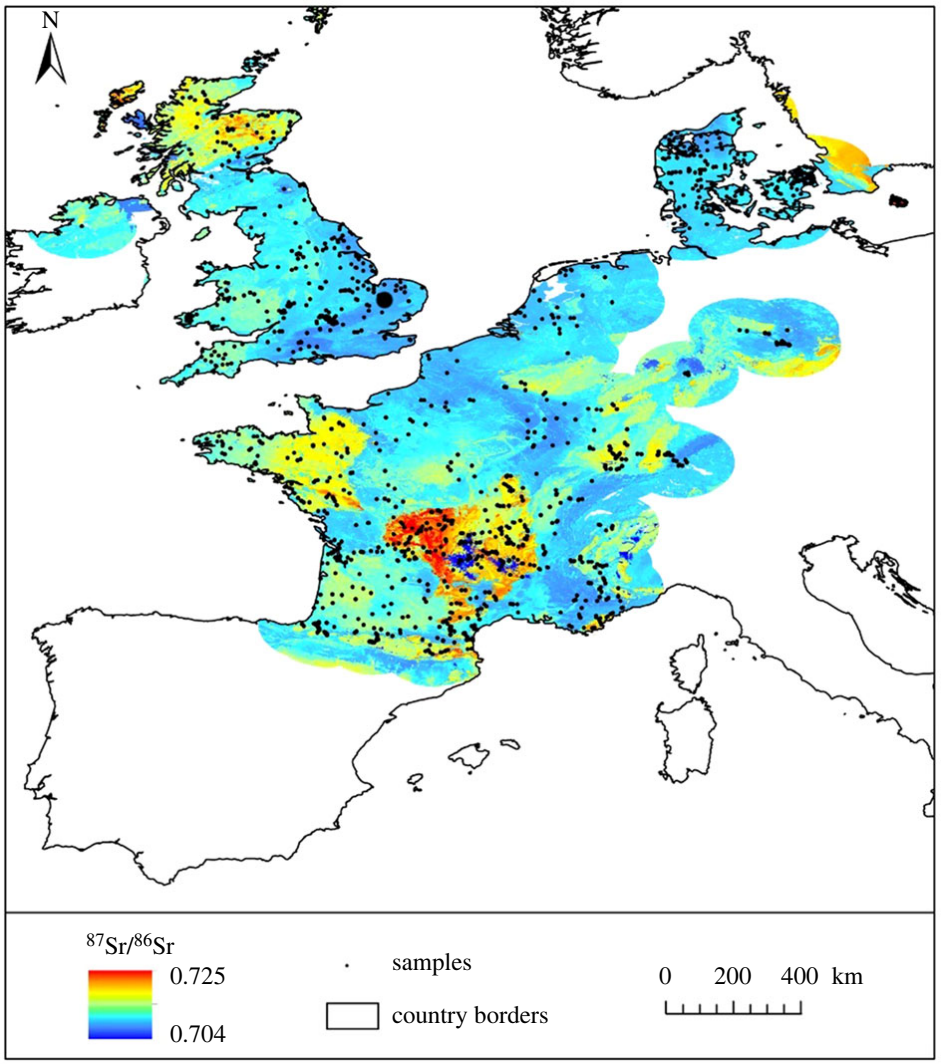
Bioavailable strontium isoscape for Western Europe, after Bataille *et al.* [23]; reproduced with permission. The site of Coldrum is illustrated as a large black circle; small black circles mark the location of biosphere ^87^Sr/^86^Sr samples.

The isotope results assist in constraining the origin of the individuals in this collection, by providing direct information about both where they obtained their childhood diet and their dietary composition. ^87^Sr/^86^Sr values of enamel from both the individuals dated to the fifth to seventh centuries AD and those that date to the fourth millennium BC exclude them having obtained their childhood diet from areas where bioavailable values exceed 0.711. Examples of areas that record bioavailable values higher than 0.711 in Europe include the south-western peninsula of England; Wales; Scotland; the Armorican Massif and Massif Central in France; Norway and Sweden (figure 4) e.g. [23,24,86–88]. Enamel ^87^Sr/^86^Sr values of the individuals in the Coldrum collection dated to the fourth millennium BC contrast with the strontium isotope ratios exhibited by other Neolithic populations that have been excavated in Wales (figure 3), at Ty Isaf and Penywyrlod [70]. They also contrast with values found in an Early Neolithic burial population excavated at Whitwell, Derbyshire. Individuals from the latter site have highly radiogenic values > 0.720, that are not consistent with the bioavailable ^87^Sr/^86^Sr range for Britain, indicating they were migrants from outside the area in which they were buried [87].

The Coldrum burial monument is located on Cretaceous West Melbury Marly Chalk and Zig Zag Chalk Formation, near to local superficial deposits of Quaternary clay, silt, sand and gravel [89]. The Gault Formation is situated approximately 0.5 km to the south; Cretaceous sandstones of the Lower Greensand (Folkestone Formation) are also located to the south, within 2 km of the site. Current biosphere mapping suggests that all the individuals sampled by this study, both those dated to the fifth to seventh century AD and those dated to the fourth millennium BC, have strontium isotope ratios that could be consistent with the locally bioavailable ^87^Sr/^86^Sr range of 0.7079–0.7103, within 2 km around the site of Coldrum [20]. Individuals in both groups could have obtained their resources locally, on lithology of the type that surrounds this site. However, ^87^Sr/^86^Sr values within this range are also bioavailable in other areas, such as south-central and eastern England and the immediately adjacent European mainland to the east, in northeastern France, Belgium and the Netherlands. e.g. [23,81,90]

The human remains in the Coldrum collection that date to the forty-first to thirty-eighth centuries BC are among the earliest to be associated with a Neolithic monument in Britain [6,7,14] and the site has therefore played a critical role in debates about how farming began in Britain e.g. [3–7]. Agriculture was already well established on the adjacent European mainland at this time and the inception of farming in Britain involved the introduction of domestic animals and plants from Europe, as well as the importation of new technologies, such as pottery manufacturing and the practice of burial monument construction [1,2]. Recent DNA evidence indicates that farming techniques and Neolithic practices were introduced by migration of populations from the immediately adjacent European mainland [91]. There are parallels between the Neolithic material culture of eastern Britain and that of northeastern France and Belgium during this period [92]. However, it is not possible to test the hypothesis of movement from such areas to southeastern England using ^87^Sr/^86^Sr or *δ*^18^O analysis (see below), as the biosphere strontium isotope values present in southeastern England are similar to those recorded in these areas of the immediately adjacent European mainland e.g. [81,93].

While some of the individuals in the current study have dates that fall within the early Neolithic, within the first two centuries of the fourth millennium BC, the mandible of one individual sampled by this study is dated to the late fourth millennium BC (3369–3106 cal BC: Maidstone Museum Specimen 6). Enamel from the second deciduous molar tooth of this individual, a child aged approximately 18 months at death, gave an ^87^Sr/^86^Sr value of 0.7096. Enamel from this individual was taken from the second deciduous molar tooth. Like the cusps of permanent first molar teeth, the crown of the deciduous second molar tooth begins to form *in utero* [26,27]. Hence it is possible the ^87^Sr/^86^Sr value of enamel reflects a contribution of dietary strontium prior to birth from resources that were exploited by the mother of the individual [54]. As with values exhibited by the other individuals sampled during this study, the strontium isotope ratio of enamel from this individual also falls within the locally bioavailable ^87^Sr/^86^Sr range around the site of Coldrum, although as noted above such a value is also bioavailable in other regions of Britain and mainland Europe.

Variation in ^87^Sr/^86^Sr values between adjacent permanent molar teeth of those individuals dated to the forty-first to thirty-eighth centuries is low, with values varying by a mean of 0.00060 ± 0.00002 (1*σ*, *n* = 6) between adjacent teeth. Variation in ^87^Sr/^86^Sr between permanent molar teeth within the group of individuals dated to the fifth to seventh century AD is also low, with values varying by a mean of 0.00013 ± 0.00049 (1*σ*, *n* = 4) between teeth. The presence of similar values in enamel from teeth that mineralize at different times during childhood could suggest that individuals obtained all their dietary resources locally throughout early life. Alternatively, individuals could have obtained their resources from different regions that have a similar bioavailable ^87^Sr/^86^Sr range, examples of which have been mentioned above.

Among the collections dated by this study to the forty-first to thirty-eighth centuries BC are several specimens attributed to Coldrum that do not have any labelling or accessions numbers (e.g. COL/UN, COL/UN8): their provenance and attribution to this particular site could therefore be questioned. However, the age of these remains is consistent with those remains of known stratigraphic context from Coldrum. As identified by Wysocki *et al.* [14], two skull fragments are securely documented as originating from the lower level of the Coldrum burial deposit and like the remains sampled by the current study these also date to the first two centuries of the fourth millennium BC. They pre-date remains from other excavated long barrows and cairns in Britain: as discussed by Whittle *et al.* [7] the dates for burials at Coldrum appear to precede more established use of long cairns and barrows elsewhere across Britain, which began at approximately 3800 cal BC.

**Figure 5.**
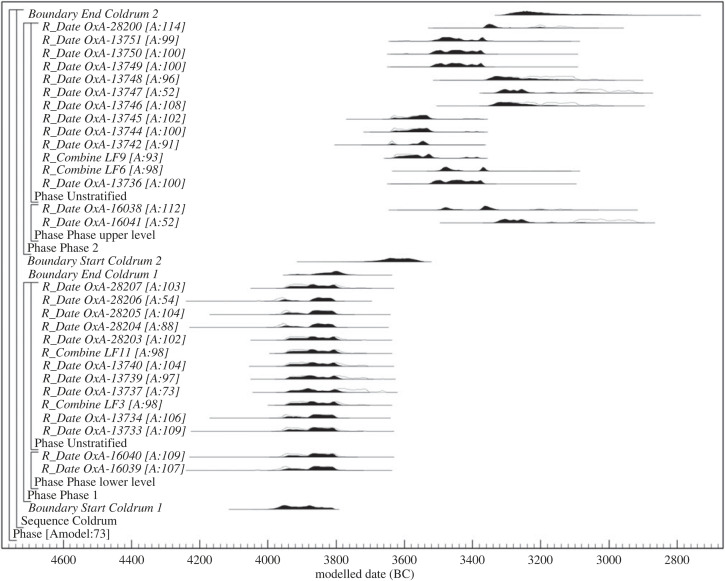
Bayesian model of radiocarbon dates from specimens of Neolithic date in the Coldrum collection, incorporating radiocarbon dates from the present study into the preferred model of Wysocki *et al*. (model 2) [14].

The presence of remains that date to both the earlier and later fourth millennium BC in the group sampled during the present study is also consistent with the hypothesis of Wysocki *et al*. [14], based on the dated remains from the site that are of documented stratigraphic context, that Coldrum was used during at least two different periods during the fourth millennium BC (see above). If dates from the present study are included within the Bayesian model of Wysocki *et al.* [14] (model 2), which incorporates the ^14^C dates for fragments of cranial vault of known stratigraphic context with those for unstratified skeletal elements attributed to the site, results suggest that early Neolithic activity began in *3980–3810 cal BC* (*95% probable*) or *3970–3860 cal BC* (*68% probable; start Coldrum 1*) and ended in *3940–3760 cal BC* (*95% probable*) or *3860–3780 cal BC* (*68% probable; end Coldrum 1*). The subsequent second phase of activity may then have occurred in the mid to late fourth millennium BC, beginning in *3720–3540 cal BC* (*95% probable*) or *3660–3570 cal BC* (*68% probable; start Coldrum 2*) and ending in *3320–2980 cal BC* (*95% probable*) or *3330–3140 cal BC* (*68% probable; end Coldrum 2*; calibrated using IntCal20 and modelled using OxCal 4.4; figure 5) [15,16].

If converted to *δ*^18^O_phosphate_, values of the second and third permanent molars of individuals dated to the fifth to seventh centuries AD range between 18.2 and 18.8‰ (mean 18.5 ± 0.2‰, 1*σ*, *n* = 4). Enamel *δ*^18^O_phosphate_ values of the second and third permanent molars of individuals who date to the forty-first to thirty-eighth centuries BC range between 17.7 and 18.3‰ (mean 18.1 ± 0.2‰, 1*σ*, *n* = 6). Deciduous and first permanent molars have been excluded from the following comparisons, due to the potential influence of breastfeeding on values in enamel from early forming teeth (see above). The *δ*^18^O values measured in enamel from the second and third permanent molars of both groups are not consistent with them having obtained their childhood diet from regions with a cold climate where individuals can exhibit *δ*^18^O_phosphate_ values below approximately 15.5‰, or *δ*^18^O_carbonate_ below approximately 24.5‰ (e.g. regions of Europe such as Scandinavia, or the Alps; figures 2 and 3) e.g. [47–53]. The values exhibited by both populations also contrast with those recorded in enamel from individuals who had migrated to temperate locations from regions with a cold climate, such as the migrant individuals of Early Medieval date excavated in Dorset, England or Dublin, Ireland (figure 2) [48–51].

The *δ*^18^O values exhibited by both the individuals who date to the fourth millennium BC and those dated to the fifth to seventh century AD are comparable to those recorded in other archaeological populations buried in north-western Europe during the Holocene. Their *δ*^18^O_phosphate_ values are consistent with the previously reported mean value of 17.7 ± 1.4‰ (2*σ*, *n* = 615) for Holocene human archaeological populations buried in Britain [59].

The *δ*^18^O values in enamel of the individuals of fourth millennium BC date are also comparable to those previously recorded among populations excavated in southern England who date to this period and who also have ^87^Sr/^86^Sr values within the local bioavailable range for this region (figure 3). For example, *δ*^18^O_phosphate_ values of second and third molar teeth from the fourth millennium BC population buried at Hazleton North, Gloucestershire, England range between 17.6 and 18.9‰ (mean 18.2 ± 0.4‰, 1*σ*, *n* = 20) [84], while in the population buried at Hambledon Hill, Dorset, England, *δ*^18^O_phosphate_ values range between 18.0 and 19.5‰ (mean 18.5 ± 0.4‰, 1*σ*, *n* = 23) [87].

Tooth enamel from populations dated to the fifth to seventh century AD in Kent have also previously recorded *δ*^18^O_phosphate_ values close to 18.0‰. Enamel from post-infant dentition (permanent premolars or second and third molars) from burials excavated from Ringlemere, Kent, has given values between 17.6 and 18.8‰ with a mean of 18.3‰ (*n* *=* 7) [60]. Similar *δ*^18^O_phosphate_ values have also been recorded in burial populations of fifth to seventh century AD date with comparable ^87^Sr/^86^Sr values in other areas of southern Britain. Examples include Eastbourne, Sussex [94], and Berinsfield, Oxfordshire [95]. However, comparable values close to 18.0‰ are also recorded in populations buried on the immediately adjacent European mainland. Values from Sannerville and Giberville, near Caen in northern France, for example, range between 17.4 and 18.2‰, with a mean of 17.9‰ (*n* *=* 11) [60]. The strontium isotope ratios in enamel from these populations in northern France are also comparable to those recorded in the present study (ibid.).

Evans *et al.* [59] suggest that populations local to the eastern side of Britain, where rainfall levels are lower, may have lower *δ*^18^O_phosphate_ (17.2 ± 1.3‰, 2*σ*, *n* = 83) than those in western Britain (18.2 ± 1.0‰, 2*σ*, *n* = 40) where there is higher rainfall. However, as discussed above, it has been shown that practices such as boiling water, brewing and stewing food alter the isotope composition of ingested fluids [44]. While *δ*^18^O analysis has been shown to be of broader utility for distinguishing between regions of cold and temperate climate, the effect of culturally mediated behaviour may as such preclude its use for distinguishing between adjacent geographical areas within a temperate region [60]. Hence, although the *δ*^18^O_phosphate_ values of the individuals who date to the fifth to seventh century AD (18.2–18.8‰, mean 18.5 ± 0.2‰, 1*σ*, *n* = 4) slightly exceed the range predicted by Evans *et al.* [59] for eastern Britain, this could be a consequence of culturally mediated behaviour that altered the isotope composition of ingested fluids, rather than an indication that the group was not local in origin to eastern Britain.

**Figure 6.**
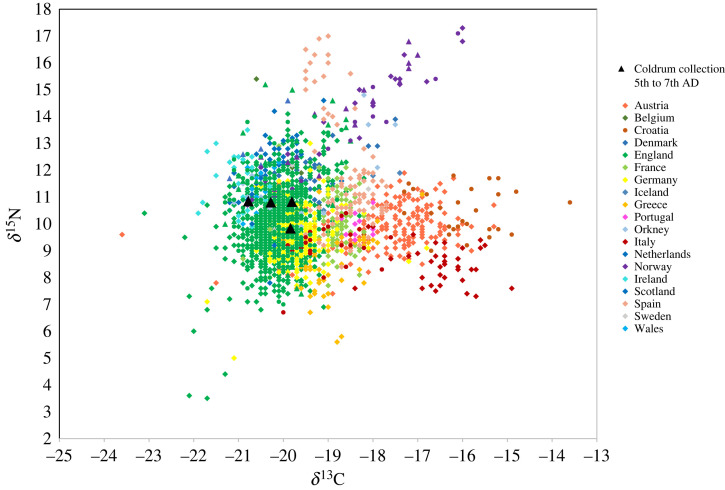
*δ*^15^N‰ (AIR) versus *δ*^13^C‰ (VPDB) values of collagen from individuals dated to the fifth to seventh centuries AD in the Coldrum collection, in comparison to populations buried in Europe during the Early Medieval period (fifth to tenth centuries AD) [98]. Triangles = first permanent molar teeth. Circles = permanent second molar, third molar and premolar teeth. Diamonds = adult bone collagen, from individuals whose skeletal age is estimated to be over 18 years at death.

**Figure 7.**
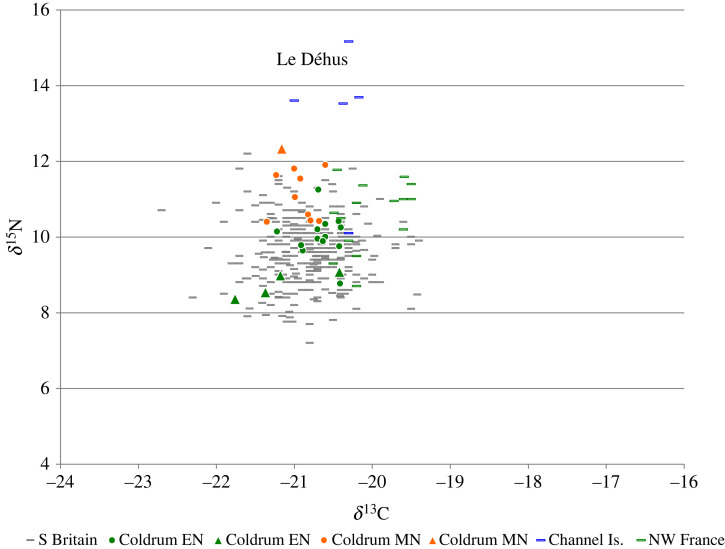
*δ*^15^N‰ (AIR) versus *δ*^13^C‰ (VPDB) values of collagen from individuals dated to the fourth millennium BC in the Coldrum collection, in comparison to populations of Neolithic date buried in England, Wales and the Channel Islands. Skeletal elements dated to the early Neolithic in the Coldrum collection = Green symbols; Mid to later Neolithic Coldrum collection = Orange symbols; Blue symbols = Le Déhus, Guernsey. Triangles = Data produced by this study; Circles = data from Wysocki *et al.* [14]. Grey dashes = Comparative data from populations buried in England and Wales during the fourth millennium BC. Data sources: [14,93,99–120].

Both the individuals who date to the forty-first to thirty-eighth centuries BC and those who date to the fifth to seventh centuries AD have *δ*^13^C_collagen_ and *δ*^15^N values that are consistent with a C_3_ terrestrial diet. In northwestern Europe values that are representative of a C_3_ terrestrial diet range between 7 to 12‰ for *δ*^15^N and −23 to −19‰ for *δ*^13^C_collagen_ e.g. [96,97]. The individuals who date to the fifth to seventh centuries AD fall within this range (figure 6), with *δ*^15^N values between 9.8 and 10.9‰ (mean 10.6 ± 0.5‰, 1*σ*, *n* = 4). Their *δ*^13^C_collagen_ values range between −20.8 and −19.8‰ (mean −20.2 ± 0.5‰, 1*σ*, *n* = 4). The individuals who date to the forty-first to thirty-eighth centuries BC also have *δ*^15^N and *δ*^13^C_collagen_ values that fall within this range (figure 7), with *δ*^15^N between 8.4 and 9.1‰, (mean 8.7 ± 0.3‰, 1*σ*, *n* = 5) and *δ*^13^C_collagen_ values between 21.8 and −20.4‰ (mean −21.2 ± 0.5‰, 1*σ*, *n* = 5). The dietary isotope results, therefore, exclude a significant contribution to diet from marine or C_4_ resources for both these groups. Both groups also have *δ*^13^C_carbonate_ values that have been shown to be typical of a C_3_ terrestrial diet, ranging between approximately −17.0 to −14.0‰ [73,74].

With the exception of Maidstone Museum specimen 6 (see above), dietary isotope analysis was conducted on bulk dentine from first molar tooth roots. The *δ*^13^C_collagen_ and *δ*^15^N results, therefore, represent the average of values that were incorporated during formation of the tooth roots. Formation of the roots of these teeth begins at approximately 3.5 ± 0.5 years and completes by approximately 7.5 ± 0.5 years of age [27]. As analysis was conducted on tooth root, rather than M1 crown dentine which begins to form earlier than this, the measured values are unlikely to have been affected by breastfeeding. Bone collagen from femora and fragments of cranial vault from adult individuals dated to the early fourth millennium BC attributed to Coldrum have previously been shown to have *δ*^13^C_collagen_ and *δ*^15^N values consistent with a terrestrial C_3_ diet (mean *δ*^13^C_collagen_ −20.7 ± 0.3‰ *n* = 10; *δ*^15^N 10.1 ± 0.7‰, 1*σ*, *n* = 10) [14]. The *δ*^13^C_collagen_ and *δ*^15^N values recorded in the forty-first to thirty-eighth centuries BC population sampled by the present study are typical of the range of dietary isotope values found in fourth millennium BC populations buried in Britain (figure 7). This is consistent with the zooarchaeological evidence from this period which indicates a diet dominated by consumption of domesticated terrestrial resources and a shift away from the routine exploitation of marine resources [104,105,121–123].

As noted above, the only individual in this study where bone, rather than M1 tooth dentine was used for dietary isotope analysis was Maidstone Museum specimen 6. This is also the only individual sampled by this study who dates to the late fourth millennium BC, to 3369–3106 cal BC (table 1). Although the individual has a *δ*^13^C_collagen_ value of −21.2‰ and *δ*^13^C_carbonate_ value of −17.0‰, typical of values within the C_3_ terrestrial range for northwestern Europe, the individual has an elevated *δ*^15^N value of 12.3‰. This individual was aged approximately 18 months of age at death, and the effect of breastfeeding may therefore explain this high *δ*^15^N value, due to the shift in trophic level that this induces [124,125]. However, as noted by Wysocki *et al.* [14], *δ*^15^N values of adult individuals in the Coldrum collection who are attributed to the same secondary phase of use during the mid to late fourth millennium BC are also high, ranging between 9.6 and 11.3‰ with a mean 10.6 ± 0.7‰, (1*σ*, *n* = 15), when compared to the mean of 9.60 ± 0.84 (1*σ*, *n* = 306) for fourth millennium BC populations buried across England and Wales (based on current data sources as cited in figure 7). *δ*^13^C_collagen_ values of individuals in the Coldrum collection (mean −20.9 ± 0.3‰, 1*σ*, *n* = 15) exclude the exploitation of marine resources as a reason for this. Wysocki *et al.* [14] suggest several possible explanations for the elevated *δ*^15^N values within the mid to late fourth millennium BC adult population from Coldrum, including consumption of manured cereal crops e.g. [126–129]; exploitation of freshwater fish e.g. [122,130], higher animal protein intake, or a greater emphasis on consumption of omnivorous species such as pigs rather than herbivores. As the site of Coldrum is located close to the Medway estuary, the exploitation of coastal marshes for animal pasture could also be a potential source for elevated *δ*^15^N values, e.g. [131,132]. In the absence of any contemporary faunal samples from the site or its environs, however, it is not possible to distinguish between alternative explanations for the elevated adult *δ*^15^N values for this period.

## 5. Conclusion

The Coldrum collection contains some of the earliest dated human remains associated with a Neolithic monument in Britain. It has therefore played a highly significant role in ongoing debate about the nature and timing of the transition to farming in Britain, e.g. [3–8]. However, this study has demonstrated that, while the collection contains human remains that date to the forty-first to thirty-eighth centuries BC, it also contains remains that date to the fifth to seventh centuries AD. The burial monument at Coldrum is of a type that is associated with of the earlier fourth millennium BC. Anglo-Saxon material culture is known from the site. There are documented examples of reuse of prehistoric monuments for burial during the Anglo-Saxon period, but the collection also has a complex curatorial history having been moved between multiple institutions in the century following its excavation. The possibility that the osteological collection from the Coldrum excavations was admixed with those from other sites prior to being transferred to the University of Cambridge following the Second World War should be considered.

The results of isotope analyses of enamel and dentine collagen assist in constraining the geographical origin of the individuals, by providing direct information as to where they obtained their childhood diet and their dietary composition. ^87^Sr/^86^Sr analysis rules out areas of radiogenic terrain (>0.711) as a source for the values that both the individuals of fifth to seventh centuries AD and those dated to the fourth millennium BC exhibit. In Europe, examples of areas that routinely record bioavailable values higher than 0.711 include the southwestern peninsula of England; Wales; Scotland; the Armorican Massif and Massif Central in France; Norway and Sweden (figure 4.) e.g. [23,24,80–82]. The ^87^Sr/^86^Sr values exhibited by both the individuals of the fifth to seventh centuries AD date and those of the fourth millennium BC are comparable to the local biosphere range (i.e. within 2 km) around Coldrum [24]. However, other areas within central southern Britain and northeastern France, the Netherlands and Belgium also have bioavailable values within the same range, e.g. [23,81,93]. *δ*^18^O values recorded in enamel from both the individuals of fifth to seventh centuries AD date and those of fourth millennium BC date are routinely found in human populations buried in temperate northwestern Europe during the Holocene (figures 2 and 3). Both groups also have *δ*^13^C_collagen_ and *δ*^15^N values that are consistent with consumption of a C_3_ terrestrial diet in northwestern Europe (figures 6 and 7). Values also exclude any significant consumption of either marine or C_4_ plant resources.

It is not possible to visually distinguish between remains in the Coldrum collection that are of fifth to seventh centuries AD date and those of fourth millennium BC date. The results of this study demonstrate how multi-isotope analysis can provide direct and independent information about the provenance of remains in museum collections, while also providing new information about diet and mobility in the past. A substantial number of archaeological sites in Britain were excavated prior to the early twentieth century. These form the basis of major archaeological collections, such as those held in the Duckworth Collection at the University of Cambridge and that of the Natural History Museum in London. This study demonstrates how the application of biomolecular techniques can assist the study of antiquarian collections such as these. Expansion in the use of these techniques will further the study of collections that were excavated over a century ago, yielding significant new information about remains whose excavation context is poorly documented, e.g. [98,133,134]. Further advances in the field of biomolecular analysis, for example expansion in use of sulfur isotopes for geographical provenancing and evaluation of dietary composition, are also likely to assist future study of such collections, e.g. [135]

## Data Availability

All data are included within the manuscript.
